# The future of psychiatry: The perspectives from a senior psychiatrist

**DOI:** 10.1192/j.eurpsy.2021.127

**Published:** 2021-08-13

**Authors:** N. Sartorius

**Affiliations:** Mental Health, Association for the Improvement of Mental Health Programs, Geneva, Switzerland

**Keywords:** Future of psychiatry

## Abstract

The future of psychiatry as a discipline (and as the main source of knowledge in the construction and functioning o mental health services) can best be grasped on the basis of an examination of the development of psychiatry over the past century in the light of current options for its functioning. Such an examination demonstrates that psychiatrists will have to expand their field of work to include the management of comorbidity of mental and physical disorders and public health approaches to the primary prevention of mental and other brain disorders. Their engagement in research will have to become restricted to psychopathology and participation in the formulation of hypotheses which will be tested in laboratory and field work;; and their involvement in teaching about mental health and illness will have to undergo a fundamental change in terms of content, methods and evaluation of effects of education which they will organize. The presentation will focus on the future tasks of psychiatrists in these areas

**Disclosure:**

No significant relationships.

